# Association between sleep duration from midlife to late life and the risk of depressive symptoms: the Singapore Chinese Health Study

**DOI:** 10.1192/bjo.2024.772

**Published:** 2024-10-11

**Authors:** Huiqi Li, Bee Choo Tai, An Pan, Woon-Puay Koh

**Affiliations:** Healthy Longevity Translational Research Programme, Yong Loo Lin School of Medicine, National University of Singapore, Singapore; Saw Swee Hock School of Public Health, National University of Singapore, Singapore; Department of Epidemiology and Biostatistics, School of Public Health, Tongji Medical College, Huazhong University of Science and Technology, Wuhan, China; Healthy Longevity Translational Research Programme, Yong Loo Lin School of Medicine, National University of Singapore, Singapore; and Singapore Institute for Clinical Sciences, Agency for Science Technology and Research (A*STAR), Singapore

**Keywords:** Sleep duration, depression, cohort study, older adults, Asian

## Abstract

**Background:**

The prospective association between sleep duration and the development of late-life depressive symptomology is unclear.

**Aims:**

To investigate sleep duration from midlife to late life in relation to risk of depressive symptoms in late life.

**Method:**

A total of 14 361 participants from the Singapore Chinese Health Study were included in the present study. Daily sleep duration was self-reported at baseline (mean age of 52.4 years; 1993–98), follow-up 2 (mean age of 65.2 years; 2006–10) and follow-up 3 (mean age of 72.5 years; 2014–16) interviews. Depressive symptoms were evaluated using the Geriatric Depression Scale at follow-up 3 interviews. Modified Poisson regression models were performed to estimate relative risks and 95% confidence intervals of late-life depressive symptoms in relation to sleep duration at baseline and the two follow-up interviews.

**Results:**

Compared with sleeping 7 h per day, a short sleep duration of ≤5 h per day at baseline (i.e. midlife) was related to a higher risk of depressive symptoms (relative risk 1.10, 95% CI 1.06–1.15), and this risk was not affected by subsequent prolongation of sleep. Conversely, a long sleep duration of ≥9 h per day at baseline was not related to risk of depressive symptoms. At follow-up 3 (i.e. late life), both short sleep (relative risk 1.20, 95% CI 1.16–1.25) and long sleep (relative risk 1.12, 95% CI 1.07–1.18) duration were cross-sectionally associated with depressive symptoms.

**Conclusion:**

Short sleep duration in midlife, regardless of subsequent prolongation, is associated with an increased risk of depression in late life. Contrariwise, both short and long sleep duration in late life co-occur with depressive symptoms.

Depressive symptoms are common in older adults and have been linked to several adverse consequences, including limited physical function, cognitive impairment and poor quality of life.^1,2^ With global ageing becoming a major problem,^3^ there is an accompanying increase in the number of older adults suffering from depressive symptoms and depression, which contribute to an increased burden of diseases.^4^ As such, there is a need to identify modifiable factors and develop risk-reduction strategies at earlier stages in life, to prevent the development of depression with ageing.

Growing evidence has suggested that given its crucial role in maintaining brain function, sleep could be a modifiable factor for prevention of late-life depression.^5,6^ In light of this, the relationship between sleep habits and late-life depression has been extensively explored, although findings from these epidemiological studies remain controversial. A meta-analysis of four prospective cohorts among older adults (>60 years) reported that neither short nor long sleep duration was significantly associated with an increased risk of late-life depression.^7^ On the other hand, more recent prospective studies demonstrated that whereas short sleep duration was associated with an increased risk of late-life depression, long sleep duration was not.^8–11^ Notably, much of the evidence is limited to cross-sectional studies or cohort studies with follow-up of <6 years. Furthermore, depression and sleep dysregulation have been identified to be reciprocally related to each other, i.e. sleep dysregulation could be a symptom of depression and is often used as a diagnostic criterion for clinical depression.^12,13^ As such, the possible reverse causality inherent in the previous studies may mask the true relationship between sleep duration and subsequent risk of late-life depression.

Furthermore, most previous studies only studied sleep duration at a single time point and did not consider changes in sleep duration with advancing age.^14^ To the best of our knowledge, only one study has investigated the association between changes in sleep duration and subsequent risk of depressive symptoms. The authors of that study, which was conducted among Korean adults, reported a higher risk of depressive symptoms related to either persistently short sleep duration or a reduction from recommended to short sleep duration.^15^ However, the interval between the last record of sleep duration and the assessment of depressive symptoms in the study was only 1–2 years, and, as shortened sleep duration could be a prodromal symptom of depression and not a risk factor *per se*, the findings could be explained by temporal bias and reverse causality.^13^ Hence, in order to conclusively investigate the longitudinal relationship between sleep duration and the development of depression in late life, it is necessary to have sufficient duration between the assessments of sleep duration and depression.

Therefore, in the present study, to determine the associations between sleep duration and the development of late-life depressive symptomology in a chronological manner, we investigated the relationship of sleep duration reported in midlife and subsequent changes over 20 years with the risk of depressive symptoms in late life in a population-based study of Chinese individuals in Singapore. We hypothesised that sleep duration and subsequent changes from midlife could influence the risk of developing depressive symptoms in late life.

## Method

### Study population

We used data from the Singapore Chinese Health Study, an ongoing prospective cohort study investigating the impact of diet, genetics and environmental factors on cancers and other chronic diseases common in Singapore.^16^ The cohort was established between 1993 and 1998 through recruitment of 27 959 men and 35 298 women, who were of Chinese ethnicity, from two major dialect groups of Hokkien and Cantonese in Singapore, and aged 45–74 years at baseline. They were either citizens or permanent residents living in government-built housing flats, where 86% of Singaporeans lived during the period of recruitment.

At enrolment, a structured questionnaire was administered in person by trained interviewers to collect detailed information on participants’ demographic factors, diet, lifestyle factors and medical history. After recruitment, consenting survivors were recontacted for follow-up 1 (1999–2004, *N* = 52 322) and follow-up 2 (2006–2010, *N* = 39 528) via telephone interviews to update selected lifestyle factors and information about comorbid conditions. During the in-person follow-up 3 interviews (2014–2016), when surviving participants were at a mean age of 73.2 years, we included assessment of ageing-related outcomes, including physical fitness, functional independence, cognitive function, depressive symptoms and quality of life. Owing to funding limitations, the follow-up 3 interviews were stopped prematurely after a total of 17 107 participants had been revisited. Participants were randomly selected for the follow-up 3 visits. In the current study, as the same question on sleep duration was asked at baseline and in the follow-up 2 and follow-up 3 interviews, only participants who completed follow-up 2 and follow-up 3 interviews were included (*N* = 17 107).

### Ethics statement

The authors assert that all procedures contributing to this work comply with the ethical standards of the relevant national and institutional committees on human experimentation and with the Helsinki Declaration of 1975, as revised in 2008. All procedures involving human subjects were approved by the Institutional Review Board of the National University of Singapore (reference code: L04-026). Written informed consent was provided by all participants. This study followed the STROBE (Strengthening the Reporting of Observational Studies in Epidemiology) reporting guidelines for cohort studies.

### Assessment of sleep duration

Sleep duration was obtained at baseline and in follow-up 2 and follow-up 3 interviews by asking the question: ‘On average, during the past year, including naps, how many hours in a day did you sleep?’ Response categories were 5 h or less, 6 h, 7 h, 8 h, 9 h and 10 h or more. Based on the results of the current analysis, sleep duration at baseline was categorised into short sleep duration (≤5 h), recommended sleep duration (6–8 h) and long sleep duration (≥9 h). As participants may have rounded up or down when reporting hours of sleep, we defined change in sleep duration as a difference of ≥2 h between baseline and follow-up 2. Using this definition, we constructed seven mutually exclusive categories representing all possible combinations of changes in sleep duration between baseline and follow-up 2.

### Assessment of covariates

At recruitment, trained interviewers used structured questionnaires to collect information about participants, including demographic factors, usual diet, cigarette smoking, alcohol consumption, physical activity, height and weight, and history of physician-diagnosed medical illnesses. Body mass index (BMI) was calculated by dividing weight (kg) by the square of height (m^2^). Physical activity was measured through self-reported data on the number of hours participants spent weekly on moderate activities, strenuous sports and vigorous work. Information on selected lifestyle factors (smoking status, alcohol intake and physical activity) and medical conditions was updated after the follow-up 2 and follow-up 3 interviews, which took place, on average, 12.4 years and 19.6 years after the baseline interviews, respectively.

In addition, we collected other ageing-related and social factors that have been linked to depression at follow-up 3, namely, self-rated health, function-limiting pain, instrumental limitations, social activity and social support.^17,18^ Participants were asked to report their self-rated health (‘excellent’, ‘very good’, ‘good’, ‘fair’ or ‘poor’) and function-limiting pain (‘no pain or discomfort’, ‘moderate pain or discomfort’ or ‘extreme pain or discomfort’). Social activity was assessed by asking participants: ‘How many hours each week do you participate in any groups (≥3 people), such as a social or work group, church-connected group, self-help group, charity, public service or community group?’ Respondents with social participation of <1 h per week were classified as having no social activity. Instrumental limitations were assessed according to eight complex activities in the Lawton Instrumental Activities of Daily Living scale,^19^ which has been validated among older adults in Singapore.^20^ Participants with at least one limitation affecting the eight activities were considered to have instrumental limitations. Social support was measured using the Duke Social Support and Stress Scale,^21^ which has previously been used to define social isolation in the current cohort.^22^ Perceived social support from family and non-family sources at different levels (‘none’, ‘some’, ‘a lot’ or ‘there is no such person’) was assessed to calculate support scores. Respondents with the lowest decile of total social support scores (range 0–100) were defined as having poor social support.^22^

### Assessment of depressive symptoms

During follow-up 3 interviews, depressive symptoms were assessed using the 15-item Geriatric Depression Scale (GDS-15). This scale, which includes 15 items with a total score ranging from 0 to 15, has been validated among community-dwelling Asian older adults. Those with total score ≥5 were considered to be depressed.^23^

Of the 17 107 participants who completed baseline, follow-up 2 and follow-up 3 interviews, we excluded 156 participants with missing values on GDS-15, 63 participants with missing values on instrumental limitations and self-rated health, and 107 participants who were blind, mute, deaf or living in nursing homes. In addition, we further excluded 2420 participants with cognitive impairment at follow-up 3, because there is evidence supporting depression being a prodrome or a consequence of dementia.^24^ Finally, 14 361 remaining participants were included in our main analyses.

### Statistical analysis

The characteristics of participants by sleep duration at baseline were compared using analysis of variance for continuous variables and Pearson's chi-squared test for categorical variables. The longitudinal associations of sleep duration at baseline, follow-up 2 and follow-up 3 with depressive symptoms were examined using mixed effects modified Poisson regression model to account for possible intra-subject correlation in repeated measures.^25^ An interaction term of sleep duration by time was included in the model to allow the effect of sleep duration to be estimated for each time point. We further used the modified Poisson regression model to examine the association between changes in sleep duration from baseline to follow-up 2 and depressive symptoms measured at follow-up 3.

Modified Poisson regression analysis directly estimates the relative risk with 95% confidence interval and is preferred over logistic regression analysis, which is used to estimate odds ratios in prospective studies, especially when the rare event rate assumption of the model is violated.^26^ Based on this approach, we constructed three models: model 1, adjusted for sex, dialect group, educational level, age and marital status at follow-up 3; model 2, further adjusted for BMI, smoking status, physical activity, alternate Mediterranean diet scores (including alcohol consumption), and self-reported history of hypertension, heart diseases, stroke and diabetes; and model 3, additionally adjusted for ageing-related factors at follow-up 3, which included instrumental limitations, function-limiting pain, self-rated health, social activity and social support. For analysis of longitudinal association between sleep duration and depressive symptoms, updated covariates at follow-up interviews, i.e. smoking status, BMI, physical activity and history of medical conditions, were adjusted as time-varying covariates in the model. To test the robustness of our analysis, we further excluded 464 subjects who reported a medical diagnosis of depression at follow-up 3 and repeated the analysis. The mixed effects modified Poisson regression model was implemented using STATA/MP version 14.0, all the other statistical analyses were conducted using SAS version 9.4, and two-sided *P*-values of <0.05 were considered to indicate statistical significance.

## Results

### Characteristics of study population

For the 14 361 participants included in the final analysis, mean ages were 52.4 ± 5.9 years at baseline, 65.2 ± 6.1 years at follow-up 2 and 72.5 ± 6.1 years at follow-up 3 interviews. Characteristics of participants by sleep duration at baseline are presented in Table 1. The proportions of participants with sleep durations of ≤5 h, 6 h, 7 h, 8 h and ≥9 h/day at baseline were 7.9%, 24.0%, 35.3%, 27.5% and 5.4%, respectively. At follow-up 2 interviews, which were an average of 12.4 years after baseline, the proportions of participants with sleep durations of ≤5 h, 6 h, 7 h, 8 h and ≥9 h per day were 11.0%, 19.2%, 27.1%, 25.3% and 17.4%, respectively, indicating increases in proportions of both short and long sleepers at follow-up 2 compared with baseline. Participants reporting sleeping ≤5 h per day or ≥9 h per day at baseline were older and more likely to be women, to be less educated, and to have stroke and diabetes compared with those who reported sleeping 7 h per day. In addition, they were also more likely to have worse general health status at follow-up 3 interviews.

**Table 1:** Characteristics of study participants at baseline (unless otherwise specified) by sleep duration^a^

Characteristics	Total	Sleep duration at baseline					*P*-value^d^
		≤5 h per day	6 h per day	7 h per day	8 h per day	≥9 h per day	
Number of participants	14 361	1131 (7.9)	3445 (24.0)	5075 (35.3)	3942 (27.5)	768 (5.4)	
Age at baseline, years	52.4 ± 5.9	53.9 ± 6.2	52.7 ± 5.9	52.3 ± 5.8	51.6 ± 5.7	52.8 ± 6.2	<0.001
Age at follow-up 2, years	65.2 ± 6.1	66.5 ± 6.3	65.5 ± 6.2	65.3 ± 6.1	64.5 ± 6.0	65.6 ± 6.1	<0.001
Age at follow-up 3, years	72.5 ± 6.1	73.7 ± 6.3	72.7 ± 6.1	72.5 ± 6.0	71.8 ± 5.9	72.9 ± 6.1	<0.001
Men	5951 (41.4)	367 (32.5)	1463 (42.5)	2129 (42.0)	1724 (43.7)	268 (34.9)	<0.001
Married at follow-up 3	10 099 (70.3)	690 (61.0)	2379 (69.1)	3628 (71.5)	2892 (73.4)	510 (66.4)	<0.001
Dialect group							0.020
Cantonese	7045 (49.1)	601 (53.1)	1679 (48.7)	2514 (49.5)	1875 (47.6)	376 (49.0)	
Hokkien	7316 (50.9)	530 (46.9)	1766 (51.3)	2561 (50.5)	2067 (52.4)	392 (51.0)	
Level of education							<0.001
No formal education	2581 (18.0)	279 (24.7)	641 (18.6)	893 (17.6)	628 (15.9)	140 (18.2)	
Primary school	6501 (45.3)	530 (46.9)	1534 (44.5)	2247 (44.3)	1795 (45.5)	395 (51.4)	
Secondary school or higher	5279 (36.8)	322 (28.5)	1270 (36.9)	1935 (38.1)	1519 (38.5)	233 (30.3)	
Sleep duration at follow-up 2							<0.001
≤5 h per day	1577 (11.0)	358 (31.7)	573 (16.6)	410 (8.1)	204 (5.2)	32 (4.2)	
6 h per day	2759 (19.2)	259 (22.9)	897 (26.0)	987 (19.5)	543 (13.8)	73 (9.5)	
7 h per day	3896 (27.1)	243 (21.5)	958 (27.8)	1546 (30.5)	1008 (25.6)	141 (18.4)	
8 h per day	3626 (25.3)	149 (13.2)	664 (19.3)	1344 (26.5)	1227 (31.1)	242 (31.5)	
≥9 h per day	2503 (17.4)	122 (10.8)	353 (10.3)	788 (15.5)	960 (24.4)	280 (36.5)	
BMI, kg/m^2^	23.1 ± 3.2	23.2 ± 3.1	23.2 ± 3.2	23.1 ± 3.2	23.1 ± 3.3	22.9 ± 3.2	0.073
AHEI score	50.9 ± 7.3	50.5 ± 7.8	50.9 ± 7.4	51.1 ± 7.1	50.7 ± 7.3	50.4 ± 7.4	0.010
Current smoker	1864 (13.0)	127 (11.2)	450 (13.1)	630 (12.4)	557 (14.1)	100 (13.0)	0.057
Physical activity, h per week^b^							0.092
<0.5	9025 (62.8)	750 (66.3)	2162 (62.8)	3154 (62.2)	2455 (62.3)	504 (65.6)	
0.5–4	3430 (23.9)	239 (21.1)	819 (23.8)	1231 (24.3)	958 (24.3)	183 (23.8)	
≥4	1906 (13.3)	142 (12.6)	464 (13.5)	690 (13.6)	529 (13.4)	81 (10.6)	
Comorbidities							
Baseline hypertension	2682 (18.7)	244 (21.6)	636 (18.5)	929 (18.3)	735 (18.7)	138 (18.0)	0.144
Baseline heart diseases	280 (2.0)	32 (2.8)	63 (1.8)	94 (1.9)	69 (1.8)	22 (2.9)	0.080
Baseline stroke	59 (0.4)	9 (0.8)	13 (0.4)	19 (0.4)	9 (0.2)	9 (1.2)	0.006
Baseline diabetes	649 (4.5)	68 (6.0)	146 (4.2)	207 (4.1)	183 (4.6)	45 (5.9)	0.023
Ageing-related factors at follow-up 3							
Depressed (defined as GDS ≥ 5)	3417 (23.8)	366 (32.4)	820 (23.8)	1145 (22.6)	891 (22.6)	195 (25.4)	<0.001
At least one limitation in IADL	3314 (23.1)	302 (26.7)	801 (23.3)	1131 (22.3)	889 (22.6)	191 (24.9)	0.018
Function-limiting pain	2775 (19.3)	277 (24.5)	681 (19.8)	969 (19.1)	684 (17.4)	164 (21.4)	<0.001
Fair or poor self-rated health	7343 (51.1)	613 (54.2)	1766 (51.3)	2603 (51.3)	1949 (49.4)	412 (53.7)	0.029
No social activity^c^	6539 (45.5)	526 (46.5)	1565 (45.4)	2288 (45.1)	1782 (45.2)	378 (49.2)	0.265
Poor social support	740 (5.2)	88 (7.8)	179 (5.2)	221 (4.4)	195 (5.0)	57 (7.4)	<0.001

BMI, body mass index; AHEI, Alternative Healthy Eating Index; GDS, Geriatric Depression Scale; IADL, Lawton Instrumental Activities of Daily Living scale.

a. Values are mean ± s.d. or *n* (%).

b. Hours per week spent on moderate activities, strenuous sports and vigorous work.

c. Social participation of <1 h per week.

d. *P*-value by analysis of variance test (continuous variables) or chi-squared test (categorical variables).

### Sleep duration and risk of depressive symptoms

During the follow-up period, 23.8% (*n* = 3417) of our participants were categorised as depressed on the basis of their GDS-15 scores. Table 2 shows the longitudinal associations of sleep duration at baseline (over an average follow-up of 19.6 years) and follow-up 2 (over an average follow-up of 7.3 years) with the risk of depressive symptoms at follow-up 3. In addition, Table 2 shows a cross-sectional association of sleep duration at follow-up 3 with concurrent depressive symptoms. In the analysis using a mixed model that accounted for timescale and time-varying confounders, short sleep duration (≤5 h per day) was associated with an increased risk of depressive symptoms at both baseline (relative risk 1.10, 95% CI 1.06–1.15) and follow-up 2 (relative risk 1.10, 95% CI 1.06–1.15) compared with the recommended sleep duration of 7 h per day. By contrast, no association was observed between long sleep duration (≥9 h per day) at either baseline (relative risk 1.01, 95% CI 0.95–1.06) or follow-up 2 (relative risk 1.03, 95% CI 0.99–1.06) and the risk of subsequent depressive symptoms. However, in the cross-sectional analysis at follow-up 3, we found that both short (relative risk 1.20, 95% CI 1.16–1.25) and long (relative risk 1.12, 95% CI 1.07–1.18) sleep durations were associated with increased concurrence of depressive symptoms. The results were consistent in the sensitivity analysis after excluding 464 participants with a self-reported history of clinical depression (data not shown).

**Table 2:** Associations between daily sleep duration and risk of depressive symptoms (*n* = 14 361)

	Cases/*N*	Model 1 relative risk (95% CI)^a^	Model 2 relative risk (95% CI)^b^	Model 3 relative risk (95% CI)^c^
Sleep duration at baseline				
≤5 h	366/1131	1.13 (1.09–1.17)	1.12 (1.08–1.17)	1.10 (1.06–1.15)
6 h	820/3445	1.02 (0.99–1.06)	1.02 (0.99–1.05)	1.01 (0.98–1.05)
7 h	1145/5075	1.00	1.00	1.00
8 h	891/3942	1.01 (0.98–1.04)	1.00 (0.97–1.03)	1.01 (0.98–1.04)
≥9 h	195/768	1.04 (0.99–1.09)	1.03 (0.97–1.08)	1.01 (0.95–1.06)
Sleep duration at follow-up 2				
≤5 h	473/1574	1.14 (1.10–1.18)	1.13 (1.09–1.18)	1.10 (1.06–1.15)
6 h	562/2759	0.99 (0.96–1.04)	0.99 (0.96–1.03)	0.98 (0.94–1.02)
7 h	837/3892	1.00	1.00	1.00
8 h	847/3622	1.02 (0.99–1.05)	1.02 (0.98–1.05)	1.00 (0.98–1.05)
≥9 h	690/2499	1.05 (1.01–1.09)	1.04 (1.01–1.08)	1.03 (0.99–1.06)
Sleep duration at follow-up 3				
≤5 h	957/2816	1.24 (1.20–1.29)	1.24 (1.19–1.29)	1.20 (1.16–1.25)
6 h	757/3599	1.08 (1.04–1.12)	1.08 (1.03–1.12)	1.07 (1.02–1.11)
7 h	594/3454	1.00	1.00	1.00
8 h	540/2591	1.08 (1.03–1.12)	1.07 (1.03–1.12)	1.07 (1.02–1.12)
≥9 h	368/1339	1.16 (1.11–1.21)	1.14 (1.09–1.19)	1.12 (1.07–1.18)

a. Model 1: adjusted for age at follow-up 3 (continuous), sex (men, women), dialect group (Hokkien, Cantonese), level of education (no formal education, primary school, secondary school or above) and marital status at follow-up 3 (married, unmarried).

b. Model 2: model 1 plus body mass index (<18.5, 18.5–22.9, 23–27.4, ≥27.5 kg/m^2^), cigarette smoking (never, former or current smoker), physical activity (<0.5, 0.5–3.9, ≥4.0 h per week), Alternative Healthy Eating Index (including alcohol drinking, continuous), medical history of hypertension, heart attack, stroke, diabetes (yes, no) at baseline or respective follow-ups as appropriate.

c. Model 3: model 2 plus instrumental limitations, function-limiting pain, self-rated health, social activity and social support at follow-up 3.

### Changes in sleep duration and risk of depressive symptoms

Given that sleep durations of 6–8 h per day were not prospectively associated with depressive symptoms in our analyses, this duration was defined as the recommended sleep duration for the subsequent analyses. Seven categories of changes in sleep duration were generated based on the baseline sleep duration and changes at follow-up 2 of the 14 361 participants who completed baseline and follow-up 2 interviews (Table 3). The proportions of participants with persistently recommended sleep duration, persistently short sleep duration and persistently long sleep duration from baseline to follow-up 2 were 62.9%, 4.3% and 3.1%, respectively. Among those with short sleep duration at baseline, 45.4% had prolonged their sleep by ≥2 h at follow-up 2, and among those with long sleep duration at baseline, 41.4% had shortened their sleep by ≥2 h at follow-up 2.

**Table 3:** Descriptions of daily sleep duration at baseline and follow-up 2 by change in sleep duration (*n* = 14 361)

Change in sleep duration		Number (%)	Sleep duration, mean (s.d.), h	
Baseline	Follow-up 2		Baseline	Follow-up 2
Recommend (6–8 h)	Difference <2 h	9033 (62.9)	7.07 (0.75)	7.13 (0.97)
	Decreased by ≥2 h	1316 (9.2)	7.45 (0.70)	5.19 (0.78)
	Increased by ≥2 h	2113 (14.7)	6.66 (0.72)	8.98 (0.78)
Short (≤5 h)^a^	Difference <2 h	617 (4.3)	5.00 (0.00)	5.20 (0.77)
	Increased by ≥2 h	514 (3.6)	5.00 (0.00)	7.84 (0.96)
Long (≥9 h)^b^	Difference <2 h	450 (3.1)	9.22 (0.41)	8.90 (0.80)
	Decreased by ≥2 h	318 (2.2)	9.46 (0.50)	6.78 (0.94)

a. There were no data on whether short sleepers at baseline further shortened their sleep by ≥ 2 h at follow-up 2.

b. There were no data on whether long sleepers at baseline further prolonged their sleep by ≥ 2 h at follow-up 2.

As shown in Table 4, there was a higher risk of late-life depressive symptoms in those reporting short sleep duration in midlife, regardless of subsequent changes at follow-up 2. Using those who persistently reported having the recommended sleep duration (6–8 h per day at baseline and change of <2 h at follow-up 2) as the reference group, we found that those with short sleep duration at baseline had increased risk of depressive symptoms at follow-up 3, regardless of their duration of sleep at follow-up 2; the relative risks (95% CIs) were 1.21 (1.07–1.36) for those with persistently short sleep duration (≤5 h per day at baseline and change of <2 h at follow-up 2) and 1.19 (1.05–1.34) for those who prolonged their sleep (≤5 h per day at baseline and lengthening of sleep by ≥2 h at follow-up 2). Those who had the recommended sleep duration at baseline but subsequently reported shorter sleep duration at follow-up 2 also had a borderline increased risk of depressive symptoms at follow-up 3 (relative risk = 1.10, 95% CI = 0.99–1.21, *P* = 0.06). By contrast, long sleep duration in midlife was not associated with risk of depressive symptoms at follow-up 3, regardless of changes at follow-up 2.

**Table 4:** Association of change in daily sleep duration from baseline (1993–1998) to follow-up 2 (2006–2010) with risk of depressive symptoms (*n* = 14 361)

Change in sleep duration		Cases/*N*	Model 1^a^	Model 2^b^	Model 3^c^
Baseline	Follow-up 2		Relative risk (95% CI)	Relative risk (95% CI)	Relative risk (95% CI)
Recommended (6–8 h)	Difference <2 h	1944/9033	1.00	1.00	1.00
	Decreased by ≥2 h	343/1316	1.15 (1.05–1.27)	1.14 (1.04–1.26)	1.10 (0.99–1.21)
	Increased by ≥2 h	569/2113	1.10 (1.01–1.19)	1.06 (0.98–1.15)	1.02 (0.95–1.10)
Short (≤5 h)	Difference <2 h	192/617	1.33 (1.17–1.50)	1.29 (1.14–1.46)	1.21 (1.07–1.36)
	Increased by ≥2 h	174/514	1.32 (1.17–1.50)	1.28 (1.13–1.45)	1.19 (1.05–1.34)
Long (≥9 h)	Difference <2 h	121/450	1.11 (0.95–1.30)	1.08 (0.93–1.26)	1.03 (0.89–1.19)
	Decreased by ≥2 h	74/318	1.06 (0.87–1.31)	1.04 (0.85–1.28)	0.98 (0.81–1.19)

a. Model 1: adjusted for age at follow-up 3 (continuous), sex (men, women), dialect group (Hokkiens, Cantonese), level of education (no formal education, primary school, secondary school or above), and marital status at follow-up 3 (married, unmarried).

b. Model 2: model 1 plus body mass index (<18.5, 18.5–22.9, 23–27.4, ≥27.5 kg/m^2^), cigarette smoking (never, former or current smoker), physical activity (<0.5, 0.5–3.9, ≥4.0 h per week), Alternative Healthy Eating Index (including alcohol drinking, continuous), medical history of hypertension, heart attack, stroke, diabetes (yes, no) at baseline.

c. Model 3: model 2 plus instrumental limitations, function-limiting pain, self-rated health, social activity and social support at follow-up 3.

## Discussion

In this prospective cohort study with longitudinal measurements of sleep duration and a long follow-up for depressive symptoms, we found that short sleep duration in midlife and later was associated with an increased risk of developing symptomatology of depression in late life, and this risk was not affected by subsequent prolongation of sleep. By contrast, long sleep duration in midlife and later was not associated with any increase in this risk. On the other hand, both short and long sleep duration in late life co-occurred with depressive symptoms and were markers of depression.

Our findings on the longitudinal relationship between short sleep duration and the development of depression were consistent with those of other prospective studies.^10,15,27^ In the ELSA study of 4545 British adults aged ≥50 years, short sleep duration (≤5 h per night) was associated with a 90% higher risk of depressive symptoms over a 6-year follow-up.^10^ Similarly, in a cohort study involving 225 915 Koreans with a mean age of 38.6 years at baseline, short sleep duration (≤6 h per night) was related to a 15% higher risk of depressive symptoms after a follow-up of 4 years.^15^ Another cohort study among 7156 Chinese adults with a mean age of 58.1 years also reported that sleep duration of <6 h per night was associated with a 45% higher risk of 2-year incident depressive symptoms.^27^ Notably, the risk estimates from these prior studies were higher than ours; one explanation for this could be that they used a logistic regression model, whereas we used a modified Poisson regression model, which tends to produce more conservative risk ratios when the rare disease assumption is not met.^26^ However, despite the modest effect size in relative risks observed in our study, the population attributable risk could still be considerable. Using our results as a basis, we estimated that late-life depressive symptoms could have been prevented in 15% of depressed participants in this cohort if all participants with short midlife sleep duration (≤5 h per day) had a sleep duration of 7 h per day instead.

Our findings of long sleep duration being a marker of but not a risk factor for depression was contrary to evidence from a meta-analysis of four cohort studies among adults aged >60 years, which reported that both short and long sleep durations were related to 15% to 40% increases in risk of depressive symptoms, respectively, although these associations were not statistically significant.^7^ However, given that the number of cohorts included was relatively small and they all had short follow-up durations (<6 years), temporal bias and reverse causality could not be confidently excluded.

The relationship between changes in sleep duration and depressive symptoms has not been well studied. To date, the only published results have been from a study of the aforementioned Korean cohort with 152 723 participants (mean age = 38.6 years).^15^ This study repeatedly measured sleep duration within 7 years and examined the subsequent 1-year risk of depressive symptoms. They found an increased risk of depressive symptoms among participants who either had persistently short sleep duration or reduced their sleep from the recommended to a short sleep duration at two time points, in line with our findings. However, this Korean study involving much younger adults also reported that the risk of depression was reduced with increased sleep duration over time. This was contrary to our finding that people who had short sleep duration in midlife but subsequently prolonged their sleep duration were still at higher risk of depressive symptoms in late life, suggesting that sleep deprivation in midlife had enduring adverse consequences that could not be mitigated by sleep prolongation in later stages of life. This is plausible, as evidence from animal studies suggests that chronic sleep restriction may lead to persistent neurobiological and neuroendocrine alterations that could in turn increase the occurrence of depression-like behaviours among rats.^28,29^ Furthermore, based on findings from another animal study, it was speculated that chronic sleep deprivation may have a reversible age threshold, with adverse effects being ameliorated in young rats, whereas frequent or chronic sleep loss in older adult rats resulted in long-lasting neurobiological changes in the brain, which included morphological alterations in the brain cortex, dysfunction in the mitochondrial activity of neurons and changes in molecular markers of brain adaptation, all of which were linked to the development and progression of poor memory retention and orienting–exploratory activities of rats that reflected gross disturbances in memory functions, emotion state and anxiety level.^30^

Several potential mechanisms have been proposed to explain the link between short sleep duration and depression. Sleep deficiency is reportedly associated with increased levels of inflammatory cytokines, such as interleukin-6, C-reactive protein and tumour necrosis factor,^31,32^ and these inflammatory markers have been shown to be predictive of depression.^33^ In addition, chronic sleep restriction may cause hyperactivity of the hypothalamic–pituitary–adrenal axis and alterations in rapid eye movement sleep, accompanied by dysregulation of monoamine neurotransmitters,^29,34^ which are known to be associated with the development of depression.^13^ Furthermore, sleep deprivation can lead to daytime sleepiness, fatigue and disrupted circadian rhythms, which have a crucial role in mood regulation.^35,36^

Our study had several strengths, including the large sample size, long follow-up duration, comprehensive data on potential confounders, and repeated measurements of sleep duration that allowed us to examine the associations between changes in sleep duration and depressive symptoms with careful consideration of time-varying confounders. However, the study also had some limitations. First, we evaluated exposure and covariates through self-report, which could not preclude non-differential measurement errors. Specifically, sleep duration was self-reported. However, we have previously published associations between sleep duration and other health outcomes such as mortality, risk of end-stage kidney disease and cognitive impairment,^37–39^ which attest to the robustness of the data on sleep duration in this cohort. Second, we did not collect information on sleep quality, such as insomnia, snoring, obstructive sleep apnoea and other sleep problems, in this study, which hampered further investigation of whether the association could be mediated or modified by these factors. Third, GDS-15 is an instrument designed for screening purposes rather than the clinical diagnosis of depression. However, this scale is feasible and widely used in large-scale population-based research studies, and its reliability has been validated.^23^ Fourth, we only measured participants’ depressed status at follow-up 3 visits; thus, we could not ascertain whether the onset preceded the earlier interviews, although they were conducted over an average of 7.3 years prior to follow-up 3. Finally, although we comprehensively adjusted for changes in lifestyle and comorbid conditions in the analysis, we acknowledge that residual confounders or changes in health and physiology due to factors not measured during follow-ups could still exist and could have biased our results. Hence, our findings should be interpreted with caution, considering the inherent limitations of an observational study.

In conclusion, our findings provide epidemiological evidence that short sleep duration in midlife, regardless of lengthening in later years, is associated with an increased risk of depressive symptoms in late life. Promotion of tailored behavioural interventions to improve sleep may have to happen at earlier life stages to reduce the risk of depression and improve quality of life in late life. These findings are particularly pertinent to preventive strategies in the general population, given the high prevalence of sleep deprivation in our modern society and its consequent far-reaching impacts on physical and mental health. Furthermore, our findings provide evidence to support initiatives that promote adequate sleep, mitigate stress-induced sleep disruptions, and improve access to affordable sleep interventions and treatments for individuals experiencing problems with sleep.

## Data Availability

The data that support the findings of this study are available from the corresponding author upon reasonable request.
